# Self-Assembly of pH-Responsive Star-Shaped Amphiphilic Polypeptides Based on l‑Lysine and l‑Leucine

**DOI:** 10.1021/acspolymersau.5c00098

**Published:** 2025-10-22

**Authors:** Daniel José da Silva, Gabriella Mendes Cobe, Raphael Colonese Vlasman, Luiz Henrique Catalani

**Affiliations:** † Institute of Chemistry, 28133 University of São Paulo, Av. Prof. Lineu Prestes, 748, CEP 05508-000 São Paulo, SP, Brazil

**Keywords:** polypeptides, rheology, secondary structure, l-leucine, l-lysine

## Abstract

Polypeptides are attractive, renewable, and biocompatible materials for broad potential biomedical, pharmaceutical, and regenerative medicine applications. In this study, star-shaped 3-arm amphiphilic polypeptides with self-assembly characteristics were synthesized by ring-opening polymerization (ROP) of *N*-carboxyanhydride (NCA), incorporating l-leucine and l-lysine to form a core–shell structure. These polymers were thoroughly characterized through various techniques, revealing that their rheological behavior and self-assembly are influenced by factors such as the length of the star-shaped arms, the proportion of hydrophobic amino acids, and the surrounding pH. The synthesized polypeptides can self-assemble into five distinct secondary structures, mimicking natural proteins. Our findings evidence that the design of the block size of the hydrophilic l-lysine core and hydrophobic l-leucine shell shapes the ability to form a physical hydrogel, exhibiting shear-thinning rheological characteristics and a rapid mechanical response. The internal microstructure of the hydrogel is based on a supramolecular self-assembly structure consisting of highly connected nanofibrils.

## Graphical abstract

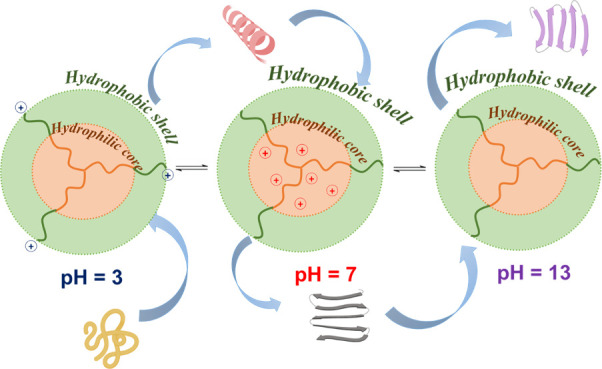

## 1 Introduction

Poly­(amino acid)­s (PAAs), also known as polypeptides, are synthetic biopolymers that feature amino acids as basic repeating units along the polymer chain.^1^ These PAAs can mimic the behavior of proteins, exhibiting the ability to self-assemble and form protein secondary structures, which are stabilized by inter- or intramolecular structures involving hydrogen bonds between the amide and carbonyl groups in amino acids.^2^ With the development of polymerization routes that yield high yields and are economically attractive, PAAs have established themselves as sustainable, functional, and structural materials to replace those derived from petroleum due to their biomass origin. Although PAAs show significant potential, their application remains limited and challenging due to an insufficient understanding of the structure–function relationship of these materials.^3^

Polypeptides can be custom-designed to meet a wide range of applications, thanks to their high versatility for chemical modification combined with their stimulus-responsive properties, biodegradability, biocompatibility, and self-assembly capacity, in addition to enabling the development of nanostructured materials.^4,5^ Among the possible applications, we highlight controlled release systems for drugs/genes/proteins (e.g., micelles, polymersomes, nanogels, microgels, nanoparticles, and nanocapsules), materials for wound healing, ultrasensitive sensors, organic-electronic and piezoelectric devices, superhydrophobic coatings, surface modification, antibiofouling surfaces, and scaffolds for regenerative medicine and tissue engineering (e.g., hydrogels, nanofibers, and porous scaffolds).^4,6–8^

The physicochemical properties of the amino acids (hydrophobicity, hydrophilicity, and positive and negative electrical charge) present in the polymer chain affect the final properties of the PAA, as observed in proteins. Furthermore, the distribution of amino acids along the polypeptide chain plays a crucial role in determining the types of self-assembled structures that biopolymers can form as well as their thermodynamic stability. The specific arrangement of hydrophilic and hydrophobic residues influences the folding and assembly behavior, ultimately affecting the stability and functionality of the resulting structures.^9–11^

Star-shaped polypeptides are PAAs with multiple linear arms radiating from a central core, offering advantages such as lower solution viscosity and enhanced functionality due to a high density of active groups.^12^ This architecture enhances solubility and flow properties, making star-shaped polymers particularly attractive for biomedical applications, such as drug delivery and antimicrobial materials.^13^

Polypeptide hydrogels are attractive for cell culture scaffold applications because their structures and properties can be easily tuned to mimic the extracellular matrix (ECM) microenvironment, making them highly biocompatible materials. Furthermore, PAA hydrogels can present rheological and stimulus-responsive characteristics suitable for 4D printing, as well as for various applications in different areas, such as food additives, superabsorbents, dressings, diagnostic devices, and others.^14,15^ Hydrogels are three-dimensional networks of chemically or physically linked hydrophilic polymer chains capable of absorbing water up to a thousand times equal to their dry mass.^16^ Physical cross-linking of hydrogels can be accomplished by thermal condensation, molecular self-assembly, chelation, or electrostatic interaction. Hydrogels can also be formed by covalent bonds, which are more stable than physical cross-linking.^17^

In the last years, amphiphilic star-shaped polypeptides from l-leucine and l-lysine, synthesized via ring-opening polymerization (ROP), have shown great promise.^18–22^ These materials form supramolecular hydrogels with shear-thinning properties, high biocompatibility, and nanofibril structures. l-Lysine-based star-shaped polymers, especially those incorporating hydrophobic residues like l-leucine or phenylalanine, demonstrate improved stability, self-assembly, and bioactivity.^23,24^ They are ideal for tissue engineering, drug delivery, and nerve regeneration and also show potential in antimicrobial and anticancer applications. Despite tailored molecular designs, a gap remains in understanding how the length of the polymer arm affects the properties of these 3-arm core–shell polypeptides, highlighting a crucial area for future research to fully realize their capabilities.

Herein, we synthesized novel star-shaped 3-arm amphiphilic polypeptides based on l-lysine and l-leucine with a pH-responsive self-assembly behavior. The results indicate that the length of the hydrophobic shell and the length of the star-shaped polymer arms affect the rheological, thermal, and self-assembly properties of the polypeptides. The polypeptides also exhibit self-assembly behavior and the ability to form a physical hydrogel that is adjustable to the size of the polymer chain. Moreover, this study provides further insights into the relationship between the structure, rheological behavior, and shear mechanical response of synthetic polypeptides in aqueous solution by utilizing rheometry and computational protocols.

## 2 Materials and Methods

### 2.1 Materials

Tetrahydrofuran (THF, HPLC grade), hexane (analysis grade), 2,5-dihydroxybenzoic acid (super-DHB, ≥99.0%), *N*(ε)-ben­zyl­oxy­carb­onyl-l-lysine (H-Lys­(Z)-OH, ≥99%), diethyl ether (analysis grade), *N*,*N*-dimethylformamide (DMF, analysis grade), alumina, tris­(2-aminoethyl)­amine (TRIS, 96%), methacrylic anhydride (containing 2000 ppm topanol A as an inhibitor, ≥94%), trifluoroacetic acid (TFA, 99%), NaOH, potassium bicarbonate (analysis grade), potassium carbonate (analysis grade), glacial acetic acid (100%), HBr (33% in acetic acid), ethyl acetate (analysis grade), anhydrous ethanol (analysis grade), molecular sieves, anhydrous methanol (analysis grade), triphosgene, phosphotungstic acid, and propylene oxide (≥99%) were supplied by Merck (Brazil). Tribasic sodium phosphate (analysis grade), dibasic sodium phosphate, and l-leucine (Leu, analysis grade) were purchased from Loja Synth S.A. (Brazil). Dialysis membranes (SnakeSkinTM, molecular weight cutoff MWCO = 3.5 kDa) were supplied by Thermo Scientific (USA). The culture media DMEM, high glucose (Dulbecco’s Modified Eagle Medium, 12800017, ThermoFisher), MEM-α with ribonucleotides (Minimum Essential Medium α, M0644, ThermoFisher), fetal bovine serum (FBS, 12657029, ThermoFisher), and trypsin-EDTA 0.25% (Trypsin-EDTA solution, T4049, Sigma-Aldrich, Merck, Brazil) and alamarBlue Cell Viability Reagent (DAL1100, Invitrogen) were used in cell culture and cytotoxicity assays.

### 2.2 Methods

#### 2.2.1 Drying and Purification of Solvents

THF, methanol, and hexane were previously left in sealed flasks and maintained with 3 Å molecular sieves for at least 24 h. The THF flask was coated with aluminum foil to protect the solvent from electromagnetic radiation. DMF was kept in a sealed flask with alumina for 24 h under light protection to avoid photodegradation of the solvent. After drying, DMF was distilled at 60 ± 5 °C under a reduced pressure of 20 mmHg.

#### 2.2.2 Drying the Reagents

Triphosgene was dried at room temperature under a low pressure of 20 mmHg for at least 12 h before *N*-carboxyanhydride (NCA) synthesis. All amino acids were dried at 60 °C and 20 mmHg for 15 h before polypeptide synthesis.

#### 2.2.3 Synthesis of H-Lys­(Z)-OH *N*-Carboxyanhydride (Lys­(Z)-NCA)

The NCA of H-Lys­(Z)-OH was synthesized by adapting a protocol previously reported in the literature.^37^ Dried H-Lys­(Z)-OH (18.38 g, 65 mmol) was suspended in 170 mL of dry THF with five 3 Å molecular sieves. Then, poly­(propylene oxide) (18 mL) was inserted into the reaction medium. Triphosgene (10 g, ∼34 mmol, 2:1) dissolved in THF (10 mL) was slowly dripped into the amino acid suspension in THF. The reaction was carried out at room temperature, with constant stirring, and under nitrogen gas flow until the reaction medium became transparent (around 3 h). The gases generated in the reaction were neutralized in an aqueous solution of NaOH (0.1 M), using a gas-washing flask coupled to a volumetric flask. Dry nitrogen gas was bubbled into the reaction medium for 30 min after NCA formation was completed to remove residual gaseous byproducts. After the reaction, the mixture was precipitated (twice) in excess anhydrous hexane in a dry ice bath. Lys­(Z)-NCA was dried in a vacuum to produce a white powder. Yield: 20.1 g (100%). Figure 1a illustrates the NCA synthetic route. NMR chemical shifts are presented in Figure S1 (Supporting Information). ^1^H NMR (300 MHz, *d*_6_-DMSO), δ_H_: 1.23–1.25 ppm (CH_2_), 1.40–1.49 ppm (CH_2_), 1.52–1.61 ppm (CH_2_), 3.0 ppm (CH_2_), 4.5 ppm (CH), 5.0 ppm (CH_2_), 7.2–7.5 ppm (benzyl), 9.1 ppm (NH).

**Figure 1:**
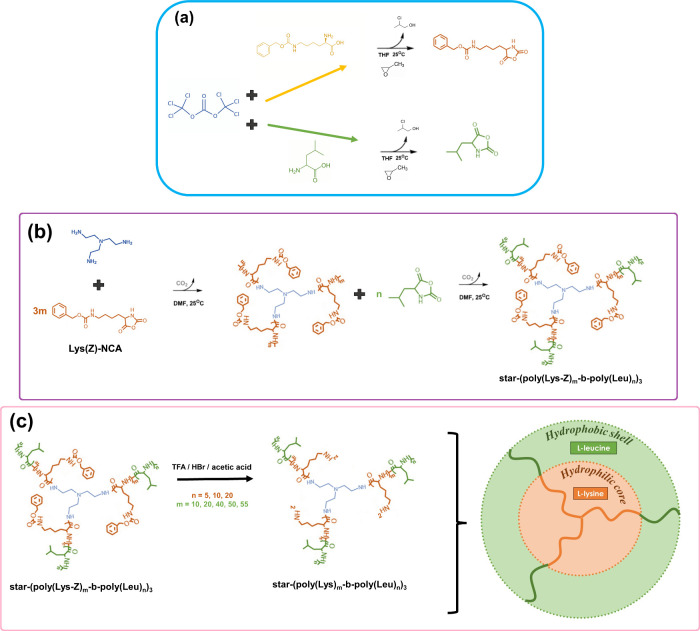
Schematics of the core–shell structure synthesis: (a) routes for NCA synthesis, (b) polymerization of star-shaped diblock polypeptides, and (c) deprotection of the star-shaped polypeptides.

#### 2.2.4 Synthesis of l-Leucine *N*-Carboxyanhydride (Leu-NCA)

l-Leucine (10.43 g, 80 mmol) was suspended in 110 mL of dry THF with five 3 Å molecular sieves. Subsequently, poly­(propylene oxide) (24 mL) was added to the reaction medium. Triphosgene (12 g, ∼40 mmol, 2:1) dissolved in THF (20 mL) was slowly dripped into the mixture. The mixture was precipitated (twice) in excess anhydrous hexane in a dry ice bath. Leu-NCA was dried in a vacuum (20 mmHg), forming a white powder as the final product. Yield: 8.3 g (65,1%). NMR chemical shifts are presented in Figure S1. ^1^H NMR (300 MHz, *d*_6_-DMSO), δ_H_: 0.8–0.9 ppm (CH_3_), 1.40–1.51 ppm (CH), 1.52–1.71 ppm (CH_2_), 4.3–4.5 ppm (CH), 5.0 ppm (CH_2_), 8.5 ppm (NH).

#### 2.2.5 Synthesis of Polypeptides with Star Diblock Architecture

Lys­(Z)-NCA (1–2 g) was dissolved in dry DMF (15 mL) with three 4 Å molecular sieves in a volumetric flask. The flask was sealed with a septum and left under constant stirring at room temperature. After the complete dissolution of the NCA, the initiator tris­(2-aminoethyl)­amine previously dissolved in dry DMF was injected. The reaction mixture was maintained for 15 h (room temperature and constant stirring). Then, the NCA of the hydrophobic amino acid (100–500 mg) was inserted into the reaction medium. The flask was sealed again, and the reaction was maintained for 3 h at room temperature with constant stirring. The reaction is schematically illustrated in Figure 1b. After the chemical reactions, the DMF was removed by rotary evaporation, and the polypeptide was precipitated in excess diethyl ether. Then, the polymer was washed with a mixture of ethyl acetate (4 mL) and ethanol (4 mL) and recrystallized in diethyl ether (25 mL) at 4 °C (24 h). The polymerization yields of the synthesized polypeptides are listed in Table S1. Figure 1b illustrates the polymerization route of the polypeptides. The NMR chemical shifts of star-(poly­(Lys-Z)_*m*_-*b*-poly­(Leu)_*n*_)_3_ are presented in Figure S2. ^1^H NMR (300 MHz, *d*_6_-DMSO), δ_H_: 0.6–0.7 ppm (CH_3_, Leu), 1.1–1.8 ppm (CH_2_, Leu+Lys-Z), 2.7–2.9 ppm (CH_2_, Lys-Z), 4.1–4.3 ppm (α-CH, Leu+Lys-Z), 5 ppm (CH_2_, Lys-Z), 7.0–7.4 ppm (benzyl, Lys-Z), 7.5–8.5 ppm (NH, Leu+Lys-Z).

#### 2.2.6 Deprotection of Polypeptides

The protected polypeptides (1–2 g) were dissolved in trifluoroacetic acid (8 mL for every 1.5 g of polymer), as illustrated in Figure 1c. Then, HBr (33% in acetic acid) was added to the solution, maintaining the ratio of 267 μL of HBr (33%) for every 100 mg of the protected polypeptide (about 1.5:1 g equivalent of HBr:polymer). After 16 h of reaction, the polymer was precipitated in diethyl ether, and the solid was washed (twice) with a mixture of ethanol (5 mL) and ethyl acetate (mL) and precipitated in excess diethyl ether. The resulting solid was dried and dissolved in ultrapure water. Finally, the solution was placed on a dialysis membrane (MWCO = 3.5 kDa) and dialyzed for 4 days by refreshing the dialysis water 3–4 times daily. Finally, the poly­(amino acid)­s were freeze-dried until completely dry. Table S2 details the prepared polypeptides and the final yields after their preparation processes. ^1^H NMR (300 MHz, *d*_6_-DMSO), δ_H_: 0.6–0.7 ppm (CH_3_, Leu), 1.1–1.8 ppm (CH_2_, Leu+Lys), 2.7–2.9 ppm (CH_2_, Lys), 4.1–4.3 ppm (α-CH, Leu+Lys).

### 2.3 Characterizations

#### 2.3.1 High-Resolution Nuclear Magnetic Resonance (NMR) Spectroscopy

^1^H NMR and ^13^C NMR spectra were recorded on a Varian INOVA 300 MHz spectrometer with deviations reported in parts per million (ppm), referenced according to the deuterated solvent peak. Measurements were carried out at room temperature (25 °C) using deuterium oxide (D_2_O) or deuterated dimethyl sulfoxide (*d*_6_-DMSO) as the solvent for PAAs and NCAs.

#### 2.3.2 Matrix-Assisted Laser Desorption–Ionization Mass Spectrometry (MALDI-ToF-ToF-MS)

MALDI-ToF-ToF-MS spectra were acquired on a Bruker MALDI Ultraflextreme instrument. The instrument was operated in reflection mode. 2,5-Dihydroxybenzoic acid (DHB) was applied as a matrix, previously dissolved in THF (20 mg mL^–1^). The poly­(amino acid)­s were dissolved in an acidic solution (acetonitrile:TFA:ultrapure water in a volumetric ratio of 70.0:0.1:29.9) at a concentration of 5 mg mL^–1^. Samples for MALDI-ToF-ToF-MS were prepared by mixing polypeptide solution with DHB solution in a volumetric ratio of 1:10.

#### 2.3.3 Size Exclusion Chromatography (SEC)

SEC measurements were conducted using a Viscotek 305 TDA Chromatograph (Malvern Instruments, UK), equipped with a precolumn and three Viscotek columns and operated with tetrahydrofuran (THF) at 35 °C. The columns included a T600 M General Mixed Org (300 × 8 mm, exclusion limit of 20,000 kg mol^–1^), an I-MBMMW 3078 (300 × 8 mm, exclusion limit of 200 kg mol^–1^), and an I-Oligo 3078 (300 × 8 mm, exclusion limit of 10 kg mol^–1^). A refractive index detector (RID) was used to monitor the results. Samples were injected in volumes of 100 μL, with a solvent flow rate set to 0.5 mL min^–1^. The SEC instrument was calibrated using polystyrene standards ranging from 500 Da to 1.21 kDa. Before analysis, polypeptide samples (10 mg) were dissolved in a THF solution (100:1 v/v) and filtered through 0.2 μm syringe filters. Table S3 lists the number-average molecular weight (*M*_n_), weight-average molecular weight (*M*_w_), and dispersity (*Đ* = *M*_w_/*M*_n_) for the synthesized polypeptides.

#### 2.3.4 X-ray Photoelectron Spectroscopy (XPS)

High-resolution XPS spectra of PAAs were collected with Kα equipment (Thermo Scientific), with monochromatic A1 Kα radiation at 10 eV for high-resolution scans at room temperature. Samples were not pretreated before XPS analyses. All XPS spectra were peak-fitted in CasaXPS version 2.3.25 with the U2 Tougaard background approximation and finite Lorentzian asymmetric line shape (LF).

#### 2.3.5 Circular Dichroism (CD) Spectroscopy

CD spectra were obtained on a JASCO J-815 spectrometer at 25 °C. Diluted polymeric solutions (0.5 mg mL^–1^) were prepared with phosphate-saline buffers (PBS, pH 7), acetate (pH 3), and bicarbonate/NaOH (pH 13). The polymer solutions were subsequently introduced into quartz cells with an optical path length of 2 mm. The CD spectra were obtained at wavelengths between 205 and 260 nm, with an integration time of 1 s, a scanning speed of 20 nm min^–1^, a number of accumulations equal to 3, and a wavelength step of 1 nm.

#### 2.3.6 Differential Scanning Calorimetry (DSC)

All DSC measurements were performed on a DSC Q10 calorimeter (TA Instruments). The polypeptides were first cooled to −50 °C and then heated to 200 °C. They were cooled back to −50 °C to remove any thermal history. Finally, the samples were reheated at 200 °C at a rate of 5 °C min^–1^. The results presented here correspond to the second heating cycle.

#### 2.3.7 Thermogravimetric Analysis (TGA)

The thermal decomposition profile of the polypeptides was identified using equipment (TA Instruments, Germany) with alumina pans. The samples were heated from 30 to 800 °C at a heating rate of 10 °C min^–1^ (N_2_ flow = 20 mL min^–1^).

#### 2.3.8 Transmission Electron Microscopy (TEM)

10 μL of the hydrogel (unprotected PAA dissolved in ultrapure water, maintaining a fixed concentration of 10% w/v and final pH 7) was dropped onto copper grids (mesh = 150). The grids were kept on qualitative filter paper throughout the sample preparation. Then, 10 μL of hydrated phosphotungstic acid solution (5% w/v in acetone) was dropped twice (every 15 min) onto the grid with the hydrogel. The sample was kept in a desiccator for 15 h before analysis in a JEOL JEM 2100 transmission electron microscope. Micrographs were obtained at an accelerating voltage of 200 kV and a magnification of 80,000×.

#### 2.3.9 Rheometry

The unprotected polypeptides were dissolved in ultrapure water, maintaining a fixed concentration of 10% w/v (final pH 7). The rheological behaviors of these polypeptide solutions were investigated on a compact modular rheometer MCR 302e (Anton Paar, Austria). The tests were carried out at 25 °C using a plate-to-plate geometry of a 25 mm diameter and a gap of 0.1 mm. Viscosity measurements were performed in the range of 10^–2^ to 10^4^ s^–1^. Amplitude sweep tests were performed at a constant angular frequency of 10 rad s^–1^ with a shear strain of 0.01 to 100%. Frequency sweep tests were conducted in the 0.1 to 100 rad s^–1^ range, using a shear strain of 0.01% (linear viscoelasticity range). The continuous weighted relaxation spectra (λ*H(λ)) and weighted retardation spectra (τ*L(τ)) were calculated with the rheological data in the linear viscoelastic range using Trios Software based on the mathematical approach proposed by Honerkamp and Weese as described in the literature.^25,26^

#### 2.3.10 Recovery Tests

Apparent viscosity recovery tests were carried out to simulate extrusion 3D printing conditions using a compact modular rheometer MCR 302e (Anton Paar, Austria). The viscosity recovery test was carried out in three intervals. A low shear rate (0.1 s^–1^) was applied in the rest interval for 10 s. Then, a constant high shear rate (100 s^–1^) was suddenly applied for 10 s. The regeneration interval was performed at a shear rate of 0.1 s^–1^ for 40 s.

#### 2.3.11 Cell Culture and Cell Viability Assays

The impact of polypeptides on cellular metabolic activity was assessed in vitro using the alamarBlue colorimetric cell viability assay. Murine hippocampal HT-22 cells and pluripotent P19 cells (from embryonic teratocarcinoma) were selected for this study. HT-22 cells were cultured in DMEM growth medium, while P19 cells were maintained in MEM-α, both supplemented with fetal bovine serum. Cells were cultured without antibiotics at 37 °C and 5% CO_2_, with the medium refreshed every 48 h.

For the assay, HT-22 and P19 cells were seeded in 96-well plates at 7 × 10^3^ and 1 × 10^3^ cells per well, respectively. After recovery, polypeptide at a concentration of 50 μg mL^–1^ (prepared in PBS) was added to the culture medium and incubated for 24 h. Resazurin dye (alamarBlue), which indicates cellular metabolic activity through redox reactions, was then added, and cells were reincubated for 4 h. Absorbance at 570 and 600 nm was measured to determine cell viability following ISO-10993-5(1) standards. Cell viability was determined according to the manufacturer’s instructions, using eq 1:

$$\mathrm{cell reduction}\;\left(\%\right)=\frac{C_{\mathrm{RED}}}{C_{\mathrm{OX}}}=100\times \frac{(\varepsilon_{\mathrm{OX}}{)}_{\lambda_{2}}A_{\lambda_{1}}-(\varepsilon_{\mathrm{OX}}{)}_{\lambda_{1}}A_{\lambda_{2}}}{(\varepsilon_{\mathrm{RED}}{)}_{\lambda_{1}}A_{\lambda_{2}}^{\prime }-(\varepsilon_{\mathrm{RED}}{)}_{\lambda_{2}}A_{\lambda_{1}}^{\prime }}$$ 1

where, *C*_RED_ is the concentration of the reduced form of alamarBlue for the test well (RED); *C*_OX_ is the oxidized form of alamarBlue for the negative control well (BLUE); *ε*_OX_ is the molar extinction coefficient of the alamarBlue oxidized form (BLUE); *ε*_RED_ is the molar extinction coefficient of the alamarBlue reduced form (RED); *A* is the absorbance of the test well; and *A*′ is the absorbance of the negative control well. The negative control well should contain alamarBlue medium but no cells. λ_1_ corresponds to 570 nm (540 nm may also be used), and λ_2_ corresponds to 600 nm (630 nm may also be used).

#### 2.3.12 Statistical analysis

All analyses of variance (ANOVA) were evaluated using GraphPad Prism 7 (Dotmatics, UK) with Tukey’s test, using a 95% confidence level.

## 3 Results and Discussion

### 3.1 Chemical Structure

The chemical characterization of polypeptides is crucial for understanding their structure, properties, and functionality, which in turn helps to identify potential applications. Here, SEC was applied to access the molecular mass distributions of the polypeptides. The dispersity (*Đ*) values close to 1.0 indicate a narrow molecular weight distribution (Table S3), which is characteristic of “living” or controlled ring-opening polymerization (ROP). In this mechanism, each polymer chain grows homogeneously from active initiator sites without significant termination or chain transfer reactions, ensuring nearly uniform chain lengths. Values of 1.04 and 1.05 reflect a high degree of control and low dispersion.

*Đ* values of 1.17–1.48, as observed in Table S3 from SEC results of the protected polypeptides, suggest a low initiation efficiency. However, such values can still be compatible with moderately controlled ROP of *N*-carboxyanhydrides. In particular, in the ROP of Lys­(Z) with tris­(2-aminoethyl)­amine, higher *Đ*s (1.17–1.48) arise because each of the tris­(2-aminoethyl)­amine initiators has three amine groups that must initiate a separate polymer chain (Figure 1b). Steric hindrance and unequal reactivity among these three initiation sites caused by the Z protecting group can reduce the effective *k*_i_/*k*_p_ ratio; *k*_i_ is the constant speed of the initiation stage (radical formation or active site), and *k*_p_ is the constant speed of the propagation stage (chain growth). This means that some arms may begin growing later than others, resulting in a broader molecular weight distribution.^27^

The chemical structure of the unprotected polypeptides was confirmed by ^1^H NMR (Figure S2). The removal of benzyloxy-carbonyl (CBZ, also named Z) groups, the Z protecting group of the amine groups on the l-lysine units, was successfully obtained through deprotection using a mixture of hydrobromic acid with TFA and acetic acid. This deprotection was confirmed by the presence of signals at 5 ppm (CH_2_, Lys-Z) and 7.0–7.4 ppm (benzyl, Lys-Z) in the ^1^H NMR spectra (Figure S3).^28^ Also, the integration signal from the repeating units of the polypeptides in ^1^H NMR spectra confirms the theoretical composition of the polymers, i.e., the designed number of repeating units on the star-shaped polymer arms. The polymerization yields of the purification processes for unprotected polypeptides (Table S2) are quite low, which is connected to the fact that the star-shaped polymers can pass through the pores of the dialysis membranes, mainly to the polymer with the lowest number of repeating units on its arms.

The MALDI-ToF-ToF-MS spectra in Figure S4 indicate the formation of l-lysine (characteristic fragments of 128 Da) polypeptides with l-leucine (characteristic fragments of 129 Da) due to the presence of molecular fragments of these amino acids and the TRIS initiator (143 Da). In the MALDI-ToF spectrum of l-lysine-based polymers, the 128 Da fragment is often observed due to the loss of water (H_2_O) from the l-lysine units.^29,30^ During the ionization and fragmentation processes, the l-lysine molecule may lose a water molecule, resulting in a fragment with a mass of 128 Da. TFA protonates part of the amino groups of l-lysine in the DHB matrix. The increase in polymer chains makes it challenging to desorb polypeptides from the DHB matrix, causing the signal intensity *m*/*z* above 3000 Da to reduce for polypeptides star-(poly­(Lys)_*m*_-*b*-poly­(Leu)_*n*_)_3_ with *m* + *n* = 60. Consequently, the molar mass distribution is slightly shifted toward lower molar masses with the increase in amino acids in the arms of the star-shaped polypeptides.

The polypeptides were analyzed using high-resolution XPS spectroscopy to assess the N_1s_ region, revealing characteristic binding energies for primary (398.9 eV), secondary (401.4 eV), and tertiary (402.2 eV) amines in Figure S5. The experimental NH_2_/NH signal ratios for polypeptides are below 0.60, indicating the formation of polypeptides with a 3-armed star architecture because their theoretical NH_2_/NH signal ratio is below 0.66.

### 3.2 Secondary Structures

The CD spectra of polypeptides dissolved in different buffers are shown in Figure 2, highlighting the effects of pH on the hydrophilic core and hydrophobic shell of the polymer. Clearly, there are changes in the profile of the CD curves as a consequence of the pH and the structure of the polymer chain. As illustrated, no amine groups are protonated at pH 13. The amine groups of l-lysine (core) are protonated at pH 7 and pH 3. At pH 3, only the terminal amines of l-leucine that form the hydrophobic shell are protonated. In these star polypeptides, the poly­(l-lysine) core carries amino groups (p*K*_a_ ≈ 10.5) that are fully protonated (NH_3_^+^) at pH 3 and 7, conferring positive charge and solubility, and deprotonate to NH_2_ above pH 10.5 (pH 13). In the l-leucine shell, α-carboxyl termini (p*K*_a_ ≈ 2.3) remain protonated (COOH) at pH 3, become largely deprotonated (COO^–^) at pH 7, and fully deprotonate at pH 13, thereby tuning the hydrophilicity–hydrophobicity balance and pH responsiveness.

**Figure 2:**
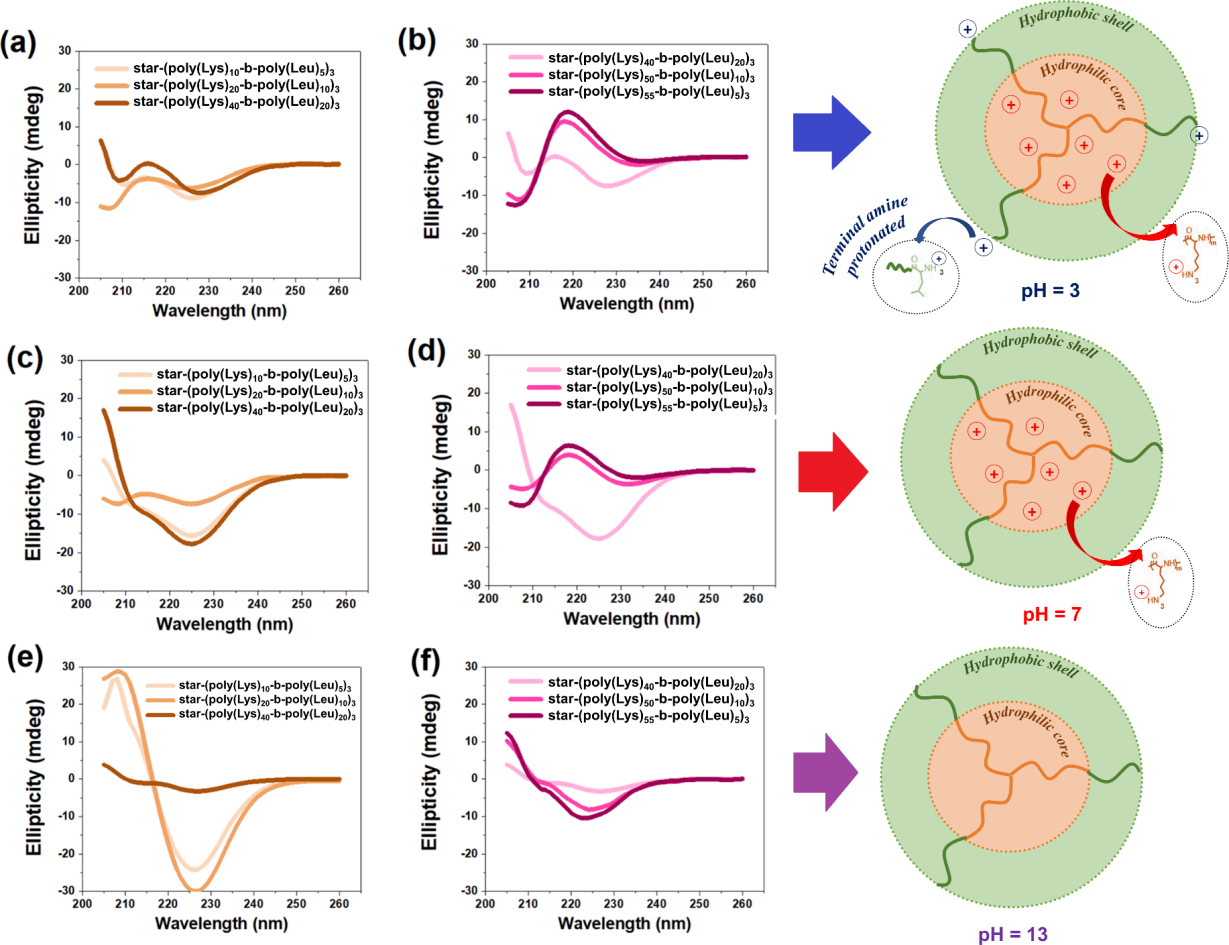
CD spectra of star-shaped diblock polypeptides in different buffers: (a, b) acetate (pH 3); (c, d) PBS (pH 7); and (e, f) bicarbonate/NaOH (pH 13).

Figure 3 shows the self-assembled secondary structures formed by the polypeptides predicted from CD spectra with the help of the BeStSel web server. The variations of the α-helix, antiparallel β-sheet, β-turn, parallel β-sheet, and random coil structures are directly associated with the protonation of the amine terminal of the amino acids distributed along the polymer chains.

**Figure 3:**
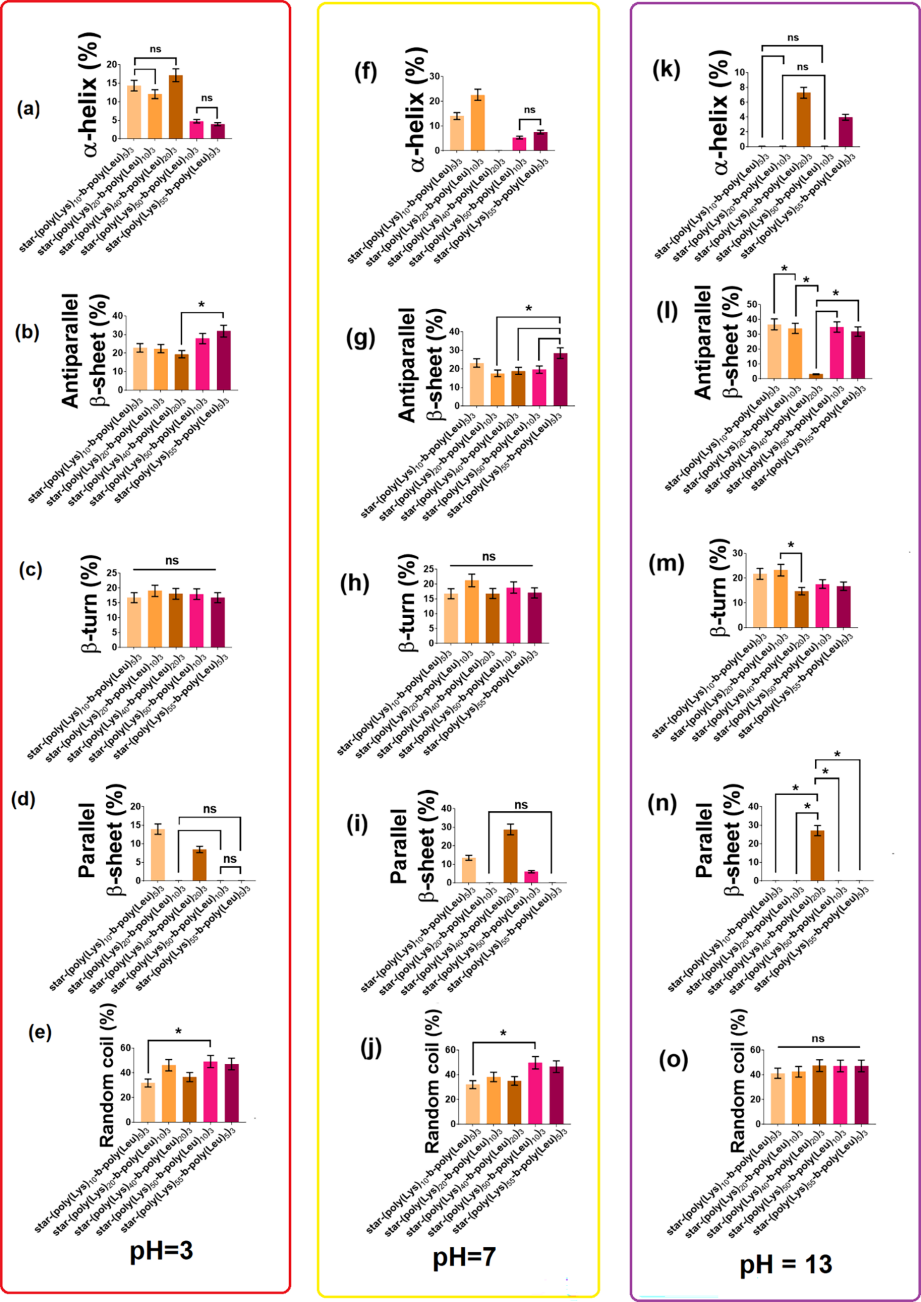
Secondary structures of the star-shaped polypeptides at different pH values: pH 3 (frame on the left), pH 7 (frame in the center), and pH 13 (frame on the right). Values are presented as mean ± standard deviation. ANOVA of significant differences between means was determined by Tukey’s test and a 95% confidence level (*p < 0.05 = significant difference) (ns = nonsignificant difference, *p* ≥ 0.05). Secondary structures were predicted from CD spectra using the BeStSel web server: (a, f, k) α-helix, (b, g, l) antiparallel β-sheet, (c, h, m) β-turn, (d, i, n) parallel β-sheet, and (e, j, o) random coil. The names of the polypeptides on the *x*-axis of the graphs are (from left to right) star-(poly­(Lys)_10_-*b*-poly­(Leu)_5_)_3_, star-(poly­(Lys)_20_-*b*-poly­(Leu)_10_)_3_, star-(poly­(Lys)_40_-*b*-poly­(Leu)_20_)_3_, star-(poly­(Lys)_50_-*b*-poly­(Leu)_10_)_3_, and star-(poly­(Lys)_55_-*b*-poly­(Leu)_5_)_3_.

The results show that increasing the polymer chain length while keeping the Lys:Leu ratio equal to 2:1 tends to reduce the relative concentration of α-helix and parallel and antiparallel β-sheet conformations. Reducing the amount of l-leucine (hydrophobic amino acid) in the polypeptide with *m* + *n* = 60 facilitates the formation of antiparallel β-sheets and α-helices but reduces the relative concentration of parallel β-sheets. The pH also affects the conformation of the polypeptides. Most polypeptides rarely form parallel β-sheets and α-helices, which indicates that these secondary structures depend on the protonation of amine groups along the polymer chain.

At pH 13, most of the l-lysine side chains (p*K*_a_ ≈ 10.5) are deprotonated, eliminating almost all positive charges and disrupting the intramolecular salt bridges that, at neutral or acidic pH, help stabilize α-helices and parallel β-sheets.^31^ This results in less “inflation” from ionic repulsion and a more uniform polymerization, favoring antiparallel β-sheets, whose hydrogen bonds are more linear and thermodynamically strong in neutral chains.^32^ The α-helix and parallel β-sheet, which rely more on hydrophobic lateral interactions and helix geometry, abruptly lose stability. In the 40 Lys/20 Leu polypeptide, the high Leu content may have created sufficient hydrophobic intermolecular interactions to maintain, even when deprotonated, lateral interactions that stabilize α-helices and parallel sheets.^33^ Thus, this composition does not follow the general pattern of an antiparallel β-sheet increase since the hydrophobic fit of the chains precisely supports the structures that, in other polymers, are undone by the loss of charges.

### 3.3 Thermal Behavior

The TGA and DTG curves are shown in Figure S6. Also, the ANOVA analysis from the TGA data is presented in Figure S6c,d. Regardless of the length of the hydrophobic block, the polypeptides show a high moisture retention capacity. The initial thermal decomposition (*T*_onset_) and maximum thermal decomposition rate (*T*_max_) are practically unmodified by the total amount of amino acids along the star-shaped polymer arms. The *T*_max_ obtained was 300 ± 5 °C. However, *T*_onset_ was significantly increased by 10 °C when the number of l-leucine units decreased from 20 to 5 in polypeptides containing 60 amino acids per arm (i.e., *m* + *n* = 60).

The thermal decomposition process of polypeptides involves two stages. The first stage occurs at around 300 °C. At this stage, the polymer backbone and its side chains begin to break down, releasing small volatile fragments such as water, carbon dioxide, and amines.^34^ This process results in the formation of a solid residue known as carbonaceous char. The second stage occurs at a significantly higher temperature, close to 550 °C. In this phase, the residual carbonaceous char, which has a more stable molecular network and is rich in aromatics, is oxidized and completely decomposed. This final step converts the remaining material into gaseous products.^35^

The DSC data in Figure S7 indicate that the glass transition temperature (*T*_g_) values for the star-shaped polypeptides are consistent, with no significant differences, averaging 21 ± 4 °C. It is well established that different factors such as the molecular weight, degree of polymerization, stereoregularity, and environmental conditions can affect this thermal characteristic of synthetic polymers.^36,37^ It is reported in the literature that the *T*_g_ is a critical feature that influences the mechanical and thermal behavior of synthetic polymers containing l-lysine as a repeating unit on the polymer chains.^38^ For example, epsilon-poly­(l-lysine) (ε-PL) typically has a *T*_g_ of around 50–60 °C, which is different from that observed for α-poly­(l-lysine) (α-PL).^30,38^ However, we find here that the amount of l-lysine and l-leucine units and molar weights did not significantly change the *T*_g_ of the polypeptides, as seen in Figure S7c.

### 3.4 Hydrogel Morphology and Rheological Behavior

The inversion tests of the polypeptide solutions (Figure 4) show that only star-(poly­(Lys)_20_-*b*-poly­(Leu)_10_)_3_ formed a hydrogel at pH 7 using a low concentration of polymer (10 wt %). As a consequence, only this sample showed a storage modulus (*G*′) that was slightly higher than the loss modulus (*G*″) in oscillatory frequency sweep tests (Figure 4), confirming the formation of the physical gel. The solutions of the other polypeptides show much *G*″ (>100 times) than *G*′ values, suggesting little mechanical resistance of these complex fluids to shear. Furthermore, the low *G*″ values (≤10^2^ Pa) indicate low resistance to flow imposed on polypeptides that did not form a physical hydrogel. The linear viscoelastic region was identified by the oscillatory amplitude sweep test (Figure S8).

**Figure 4:**
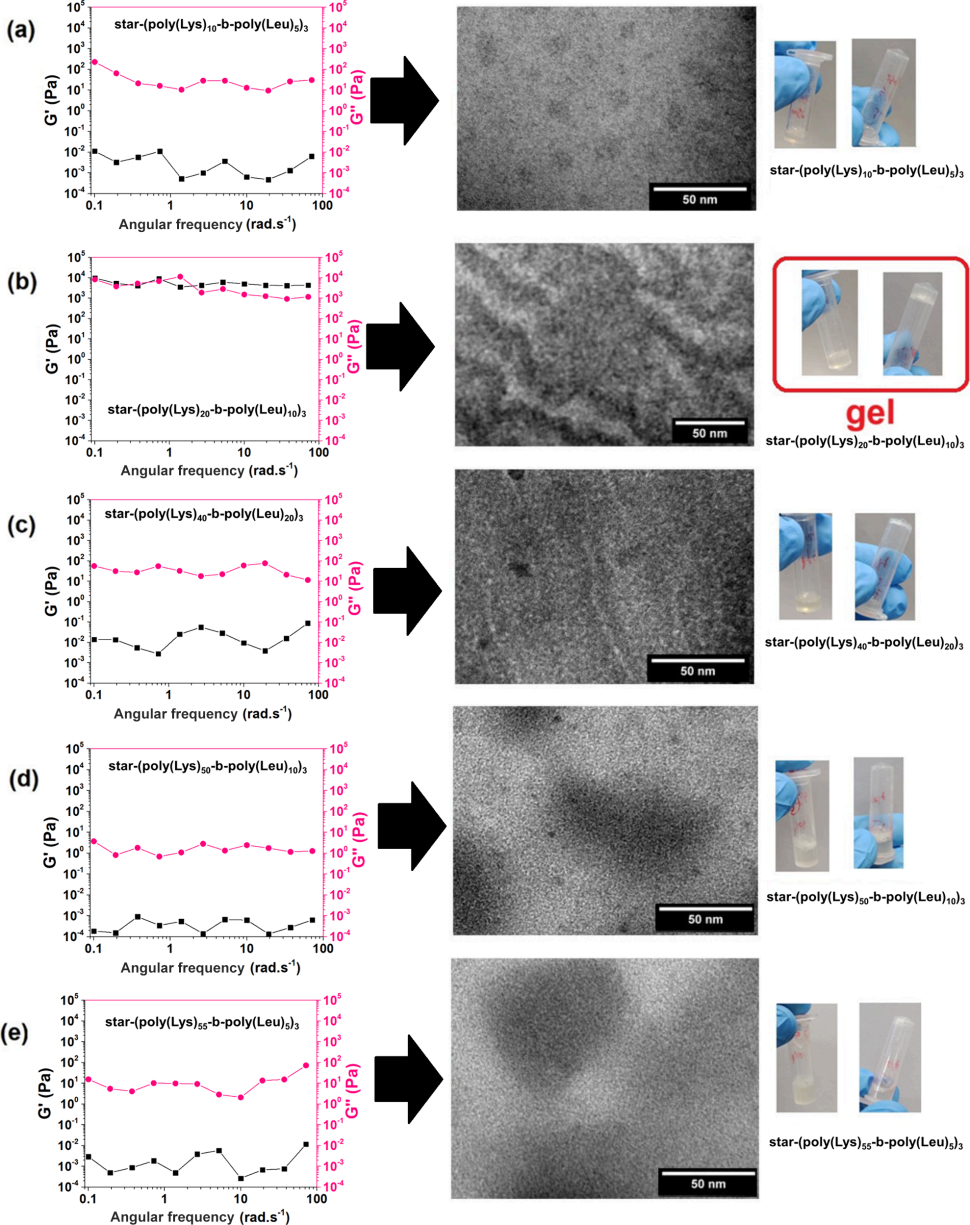
TEM images and storage (*G*′) and loss (*G*″) modulus curves of the polypeptides in water (pH 7 and temperature = 25 °C) obtained by oscillatory frequency sweep tests: (a) star-(poly­(Lys)_10_-*b*-poly­(Leu)_5_)_3_, (b) star-(poly­(Lys)_20_-*b*-poly­(Leu)_10_)_3_, (c) star-(poly­(Lys)_40_-*b*-poly­(Leu)_20_)_3_, (d) star-(poly­(Lys)_50_-*b*-poly­(Leu)_10_)_3_, and (e) star-(poly­(Lys)_55_-*b*-poly­(Leu)_5_)_3_. Inversion tests of aqueous solutions (pH 7 and temperature = 25 °C) of the star-shaped polypeptides are presented: the star-shaped polypeptide solution that formed a physical gel is highlighted with a red square.

The TEM images in Figure 4 evidence that the gel formation is connected to a self-assembly behavior that originates a microstructure in the star-(poly­(Lys)_20_-*b*-poly­(Leu)_10_)_3_ hydrogel solution that is formed by highly connected self-assembled nanofibrils forming a network, as evidenced in other polypeptides.^39,40^ Consequently, the star-(poly­(Lys)_20_-*b*-poly­(Leu)_10_)_3_ solution showed the highest apparent viscosity compared to the other polypeptide solutions (Figure 5a,b). All samples exhibited a non-Newtonian fluid behavior of the shear-thinning type, which is attributed to the breakdown of the fluid’s internal structure as the shear rate increases. Polypeptides with longer polymer chains (*m* + *n* = 60) showed the presence of a second Newtonian plateau at frequencies above 100 s^–1^. This behavior was not observed in solutions of smaller polypeptides. The star-shaped polypeptides with arms of 15 and 30 amino acids showed the highest apparent viscosities in the shear rate ranges of 0.1 and 100 s^–1^.

**Figure 5:**
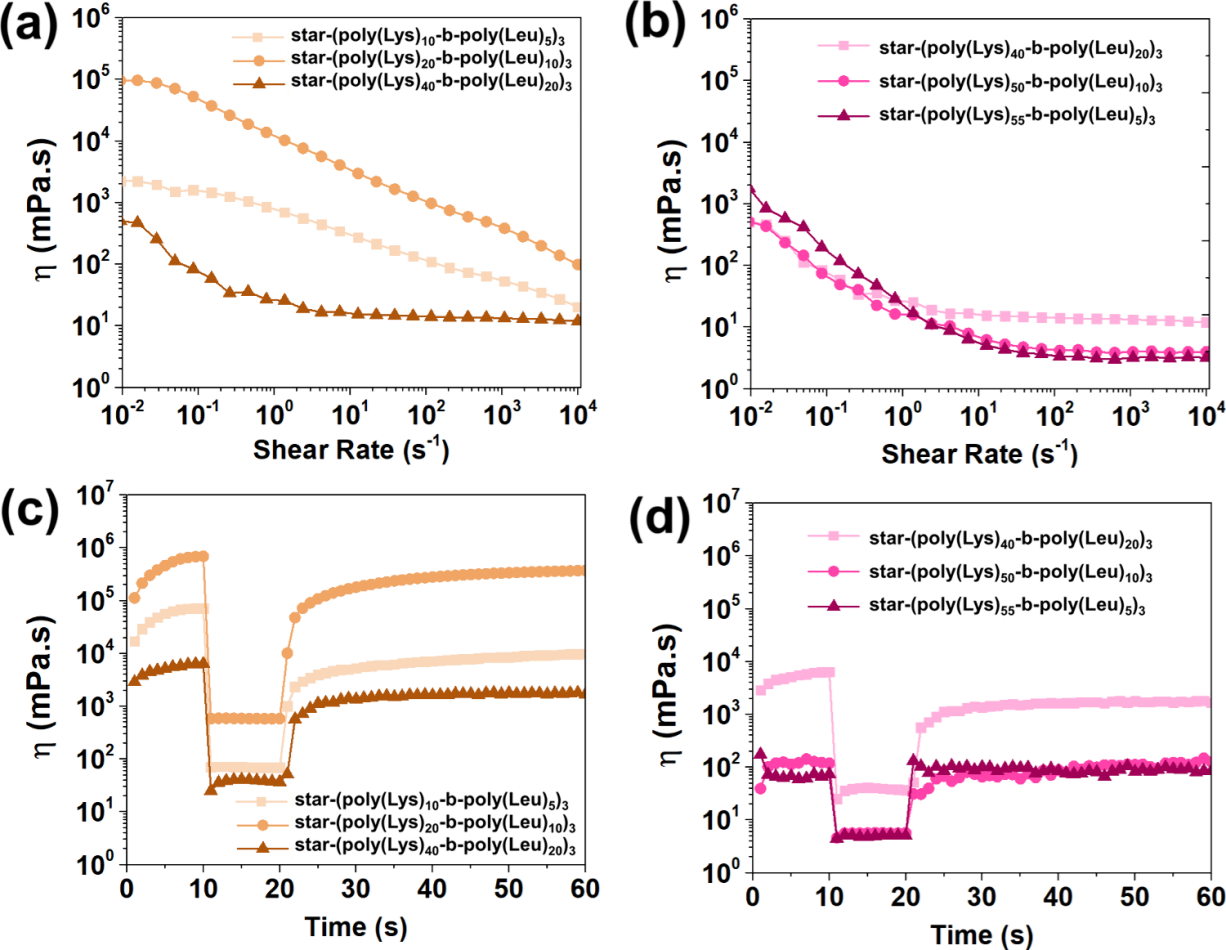
Apparent viscosity curves (η) and recovery data from polypeptides in water (pH 7 and temperature = 25 °C): (a, c) star-shaped polypeptides with different amounts of amino acids in the polymer-star arms and (b, d) star-shaped polypeptides with varying quantities of l-leucine in their hydrophobic blocks.

The shear-thinning behavior of polypeptide solutions was characterized by the consistency index (*K*) and power-law index (*n*) using power-law fitting (Figure S9). Solutions of the polypeptides with polymer arms containing 60 amino acids display the lowest *n* values, suggesting that macromolecular disentanglement under shear readily occurs when the polypeptides display 15 and 30 amino acids per polymer arm. Notably, the star-(poly­(Lys)_20_-*b*-poly­(Leu)_10_)_3_ solution showed the highest *K* value, which was attributed to its hydrogel physical network, as observed by TEM analysis.

The low- and high-shear ramps in the recovery tests of the polypeptide solutions, presented in Figure 5c,d, reveal that the samples with a self-assembled nanofibril microstructure, such as star-(poly­(Lys)_20_-*b*-poly­(Leu)_10_)_3_ and star-(poly­(Lys)_40_-*b*-poly­(Leu)_20_)_3_, take longer to recover their initial internal microstructures after submitting to the high shear rate step. This observation is attributed to the more complex self-assembly structure of the nanofibrils in these samples, which involves both molecular and supramolecular levels of structural organization. Such intricate situations tend to be kinetically slower under identical environmental conditions.^41,42^

The weighted relaxation spectra (Figure S10a) indicate that the relaxation processes of the star-shaped polypeptide star-(poly­(Lys)_55_-*b*-poly­(Leu)_5_)_3_ in polymer solutions involve times shorter than 4 ms, while the other polypeptides present faster macromolecule relaxation processes (<2 ms) as a consequence of their smaller arms that facilitate the shear stress relaxation processes caused by the physical contact of the macromolecules. The weighted retardation spectra (Figure S10b) also indicate that this star-(poly­(Lys)_55_-*b*-poly­(Leu)_5_)_3_ solution also has a longer time for shear stress retardation to occur than the other polypeptide solutions, whose retardation processes are also very fast (<2 ms). Interestingly, the aqueous solution of star-(poly­(Lys)_20_-*b*-poly­(Leu)_10_)_3_ polypeptide, which forms a physical hydrogel, presents lower signal intensities in the weighted retardation spectra compared to other polymer solutions, indicating that the formation of a three-dimensional network of this polypeptide makes its polymer solution respond quickly to mechanical stresses, even those of low magnitude, which is characteristic of very soft hydrogels.

### 3.5 Cytotoxicity

The P19 and HT-22 cell lineages are sensitive and frequently used in cytotoxicity testing because they can reflect detailed cellular responses to different materials by in vitro assays. P19 is suitable for toxicity studies in developing cells, while HT-22 is better suited for oxidative stress testing, providing a broad range of responses in a single study.^43^ The polypeptides do not display cytotoxicity against the HT-22 cell lineage, as shown in Figure 6. However, star-(poly­(Lys)_40_-*b*-poly­(Leu)_20_)_3_ and star-(poly­(Lys)_55_-*b*-poly­(Leu)_5_)_3_ reduced the P19 cell viability to below 70%, indicating cytotoxicity in this case. We chose 50 μg mL^–1^ as the test cytotoxicity concentration because it is widely used in biocompatibility studies of alginate and CMC in mammalian cells, demonstrating the absence of cytotoxic effects up to 100 μg mL^–1^.^44,45^ This dose corresponds to the upper range of effective concentrations in in vitro biomedical formulations, allowing for a conservative assessment of cellular safety.^46^

**Figure 6:**
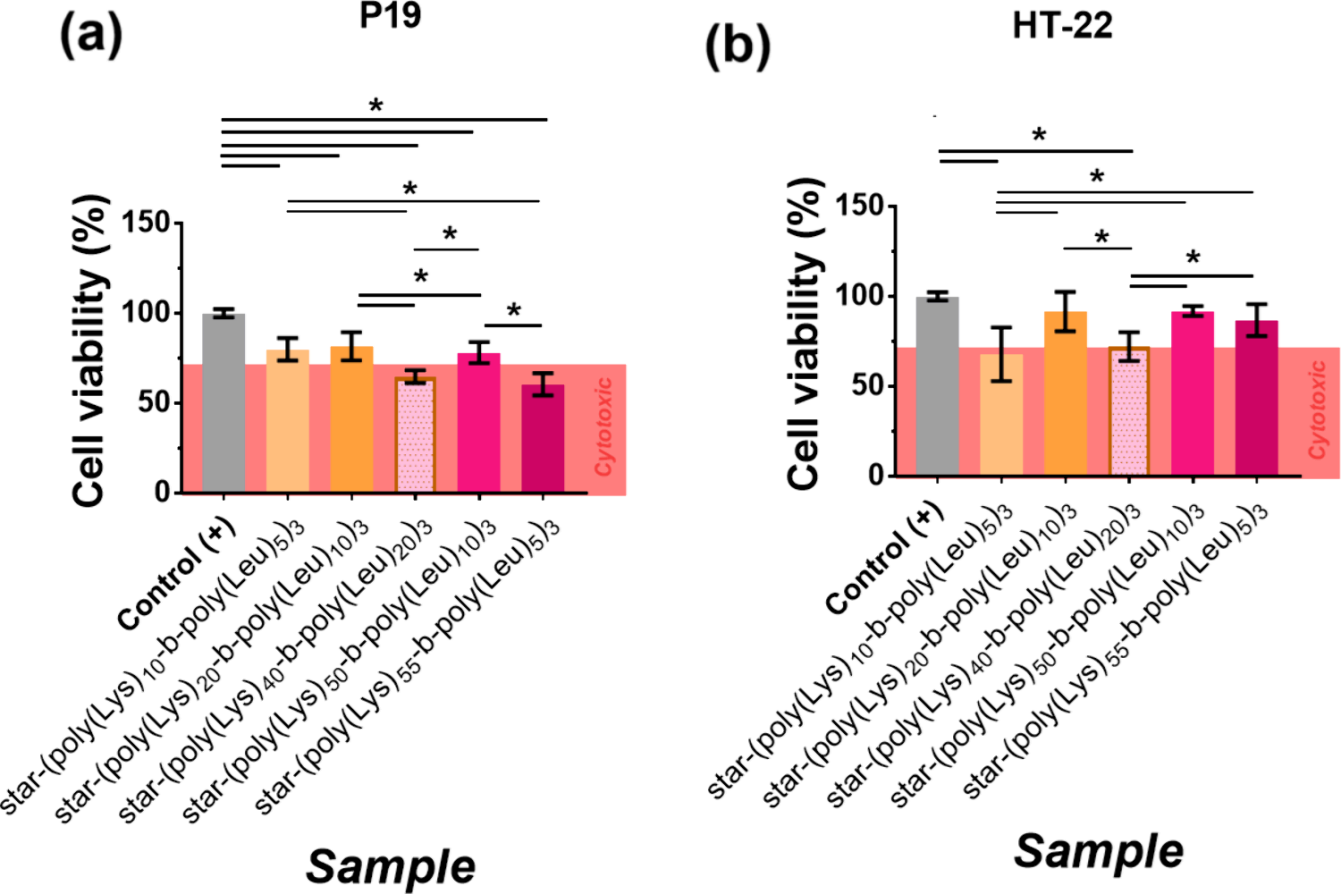
Cell viability of the star-shaped diblock polypeptides based on l-leucine and l-lysine for (a) HT-22 and (b) P19 cell lines at a polypeptide concentration of 50 μg mL^–1^. Values are presented as mean ± standard deviation. ANOVA of significant differences between means was determined by Tukey’s test and a 95% confidence level (*p < 0.05 = significant difference).

The cytotoxicity of polyelectrolytes, particularly cationic ones such as poly­(l-lysine), is closely linked to their charge properties. Cationic polyelectrolytes can interact with cell membranes due to their positive charge, potentially causing cell damage or death. Higher molecular weights and charge densities tend to enhance membrane binding and cytotoxicity.^47^ As reviewed by Zhu et al., the positive charges in polypeptides also confer bactericidal properties to these cationic polyelectrolytes, including polypeptides based on l-lysine and l-leucine, while cationic segments bind the anionic bacterial membrane through ionic interaction with the hydrophobic amino acids, facilitating their internalization into the bacterial cell through the cell membrane.^48^

The literature on the cytotoxicity of polypeptides based on l-lysine and l-leucine is still scarce. Zhang et al. identified that increasing the length of the poly­(l-leucine) block in linear block copolymers with poly­(l-lysine) favors their cellular internalization when complexed with pDNA. These polymers did not show cytotoxicity against COS-7 cells (a cell line derived from African green monkey kidney cells) when very low concentrations of these polymers were used in MTT assays.^28^ Other studies have demonstrated that increasing the poly­(l-leucine) block in diblock copolymers containing a poly­(l-lysine) block favors helix formation and improves bacterial activity. Additionally, anionic linear diblock poly­(l-glutamate)-*block*-poly­(l-leucine) showed lower toxicity in epithelial (T84) and endothelial (HULEC-5A) cell lines when compared with polypeptide systems from l-lysine with l-leucine.^49^

## 4 Conclusions

Amphiphilic star-shaped three-arm polypeptides were successfully synthesized by ROP of NCAs of l-lysine and l-leucine. These polypeptides present self-assembly and rheological behavior dependent on pH, chain size, and the amount of l-leucine in the hydrophobic block. The thermal stability of the polypeptides was affected only by the reduction of l-leucine in the chain. These polypeptides are capable of self-assembling in water into various secondary structures like proteins, including α-helices, antiparallel β-sheets, random coils, β-turns, and parallel β-sheets. Additionally, the polypeptides form polymer aqueous solutions that exhibit shear-thinning behavior at pH 7. Star-(poly­(Lys)_20_-*b*-poly­(Leu)_10_)_3_ is the only polypeptide that can form a physical gel in water at low concentrations and does not display cytotoxic effects on HT-22 and P19 cell lines, suggesting promising properties for the development of 3D printable hydrogel-based bioinks.

## Supplementary Material



## Data Availability

The supplementary results are available in the Supporting Information. Data supporting this study will be provided upon request.

## Notes

Polypeptides and polymerization yields; purification yields after deprotection; SEC data including *M*_n_, *M*_w_, and *Đ*; ^1^H and ^13^C NMR spectra of the NCAs and the protected and unprotected polypeptides; MALDI-ToF-ToF-MS spectra of the star-shaped diblock polymers; high-resolution XPS spectra in the N_1s_ region; TGA/DTG and DSC curves for different compositions of hydrophobic arms and blocks; storage (*G*′) and loss (*G*″) moduli obtained by oscillatory testing at pH 7 and 25 °C; consistency indices (*K*) and power-law exponents (*n*) of the polymer solutions; and weighted relaxation and weighted retardation spectra (PDF)
