# Supplementary material for: Self-Assembly of pH-Responsive Star-Shaped Amphiphilic Polypeptides Based on l‑Lysine and l‑Leucine
Source: ACS Polym Au. 2025 Oct 22;5(6):907–18. doi: 10.1021/acspolymersau.5c00098 (PMC12874153; doi:10.1021/acspolymersau.5c00098)
Supplement: Supplementary file 1 [file lg5c00098_si_001.pdf]

(Supplementary material)

Self-Assembly of pH-Responsive Star-Shaped Amphiphilic Polypeptides Based on L-Lysine and L-Leucine

Daniel José da Silva[1], Gabriella Mendes Cobe[1], Raphael Colonese Vlasman[1], Luiz Henrique Catalani[1*]

[1] Institute of Chemistry, University of São Paulo, Av. Lineu Prestes, 748, CEP 05508-000, São Paulo, SP, Brazil.

*** catalani@usp.br**

1. **Materials and methods**

1.1. Polypeptide synthesis

Table S1. Polypeptides and polymerization yields.

Polypeptide	Initial amount of Lys(Z)-NCA (g)	Initial amount of Phe-NCA (g)	Final product (g)	Monomers /initiator ratio - [M]/[I] (mol/mol)	Yield (%)
star-(poly(Lys-Z)$_{10}$-b-poly(Leu)$_{5}$)$_{3}$	2.03	0.09	1.27	45	66.09
star-(poly(Lys-Z)$_{20}$-b-poly(Leu)$_{10}$)$_{3}$	1.85	0.19	1.48	90	80.84
star-(poly(Lys-Z)$_{40}$-b-poly(Leu)$_{20}$)$_{3}$	1.48	0.38	1.66	180	100.00
star-(poly(Lys-Z)$_{50}$-b-poly(Leu)$_{10}$)$_{3}$	1.85	0.07	1.74	180	100.00
star-(poly(Lys-Z)$_{55}$-b-poly(Leu)$_{5}$)$_{3}$	1.53	0.19	1.16	180	75.38

Table S2. Yields of the purification processes of unprotected polypeptide.

Deprotected polypeptide	Initial amount (g) – before deprotection	Final amount (g) – after deprotection	Final amount (g) - After dialysis and freeze drying	Final yield (%)
star-(poly(Lys)$_{10}$-b-poly(Leu)$_{5}$)$_{3}$	0.998	0.907	0.303	3.43
star-(poly(Lys)$_{20}$-b-poly(Leu)$_{10}$)$_{3}$	1.174	0.862	0.341	39.56
star-(poly(Lys)$_{40}$-b-poly(Leu)$_{20}$)$_{3}$	1.853	1.406	0.145	10.32
star-(poly(Lys)$_{50}$-b-poly(Leu)$_{10}$)$_{3}$	1.398	1.364	0.470	34.44
star-(poly(Lys)$_{55}$-b-poly(Leu)$_{5}$)$_{3}$	1.110	1.543	0.425	27.52

2. Results

2.1. SEC

Table S3. SEC results for the star-shaped polypeptides based on L-leucine and L-lysine(Z).

Polypeptide	M_n (kDa)	M_w (kDa)	Dispersity (Đ)
star-(poly(Lys-Z)$_{10}$-b-poly(Leu)$_5$)$_3$	44.67	66.22	1.48
star-(poly(Lys-Z)$_{20}$-b-poly(Leu)$_{10}$)$_3$	60.68	63.19	1.04
star-(poly(Lys-Z)$_{40}$-b-poly(Leu)$_{20}$)$_3$	81.36	95.23	1.17
star-(poly(Lys-Z)$_{50}$-b-poly(Leu)$_{10}$)$_3$	80.08	96.46	1.20
star-(poly(Lys-Z)$_{55}$-b-poly(Leu)$_5$)$_3$	91.65	96.36	1.05

2.2. *RMN*

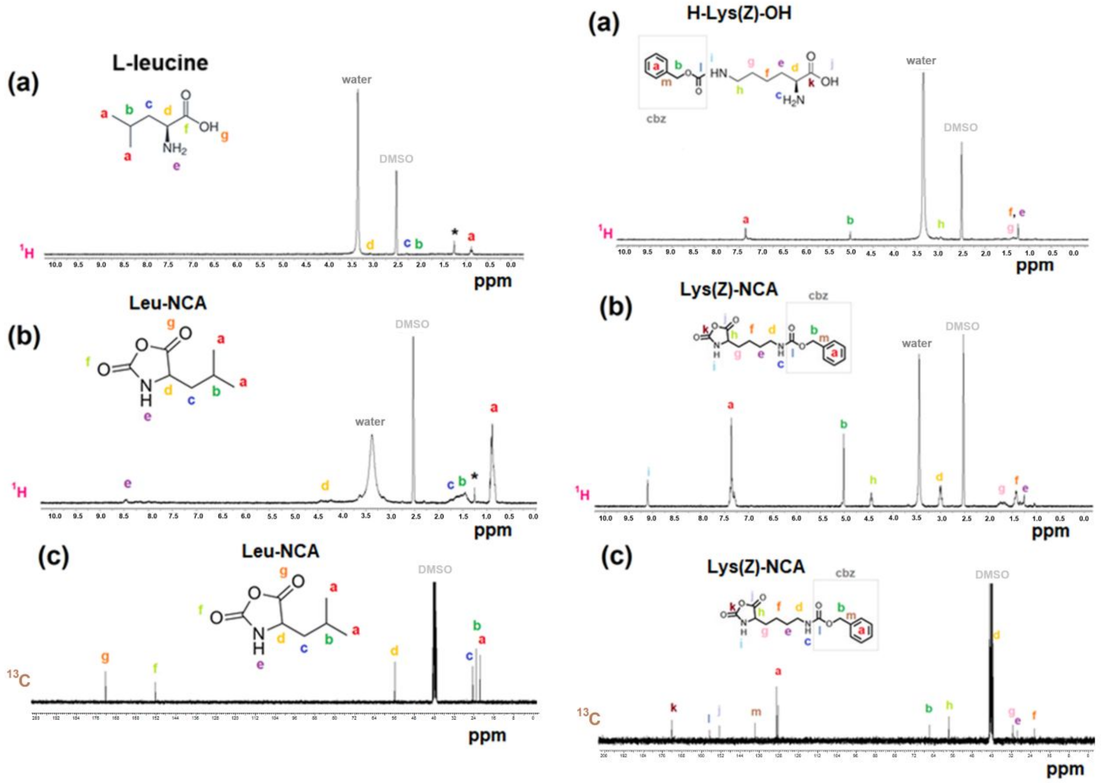

Figure S1. ^{1}H-NMR spectra from: (a) amino acids, and (b) and NCAs of the amino acids (b). ^{13}C-NMR spectrum from the the NCA of the L-leucine and L-Lysine(Z)-OH (c).

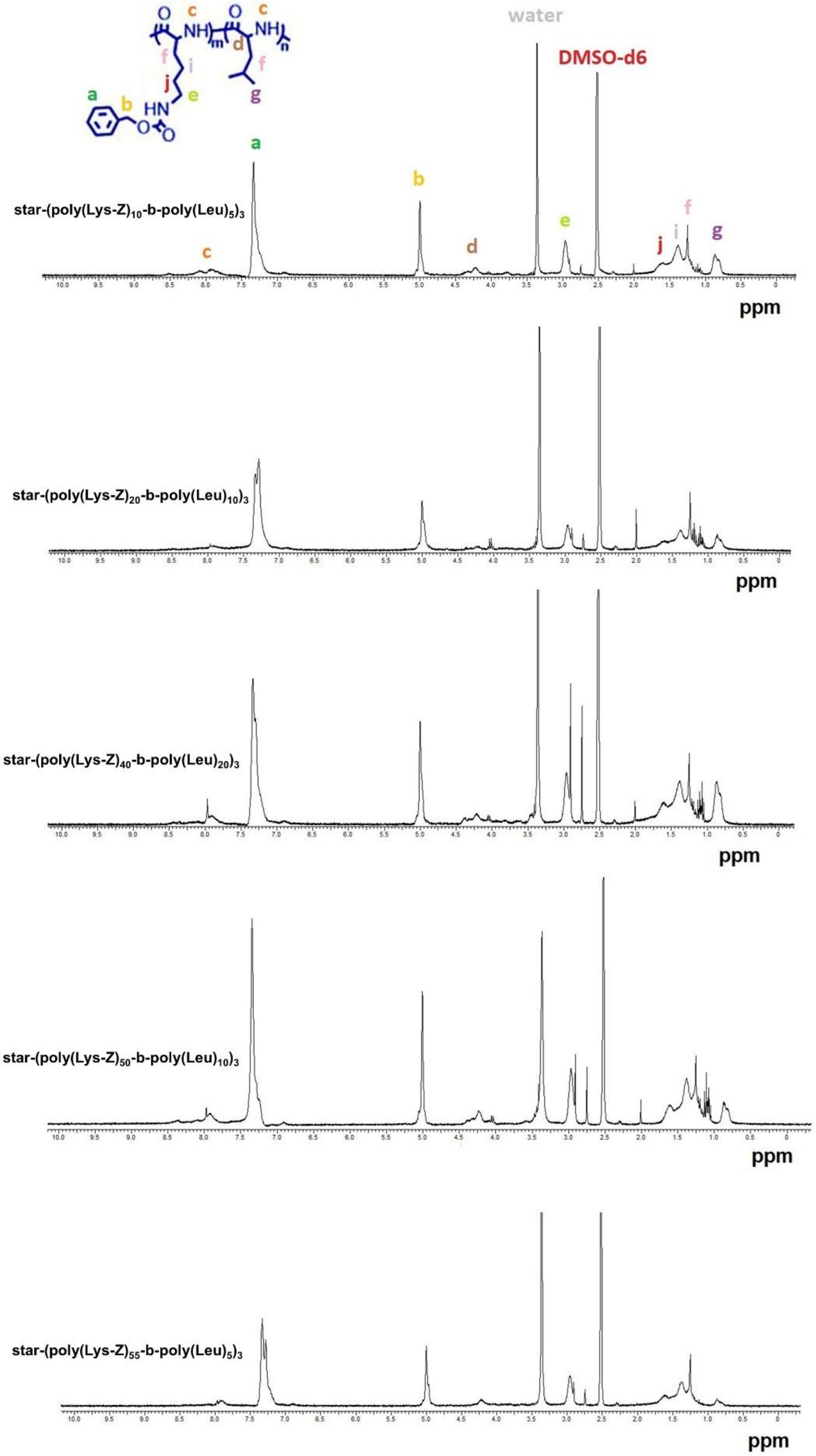

Figure S2. ^{1}H-NMR spectra of the star-shaped protected polypeptides in DMSO-d6.

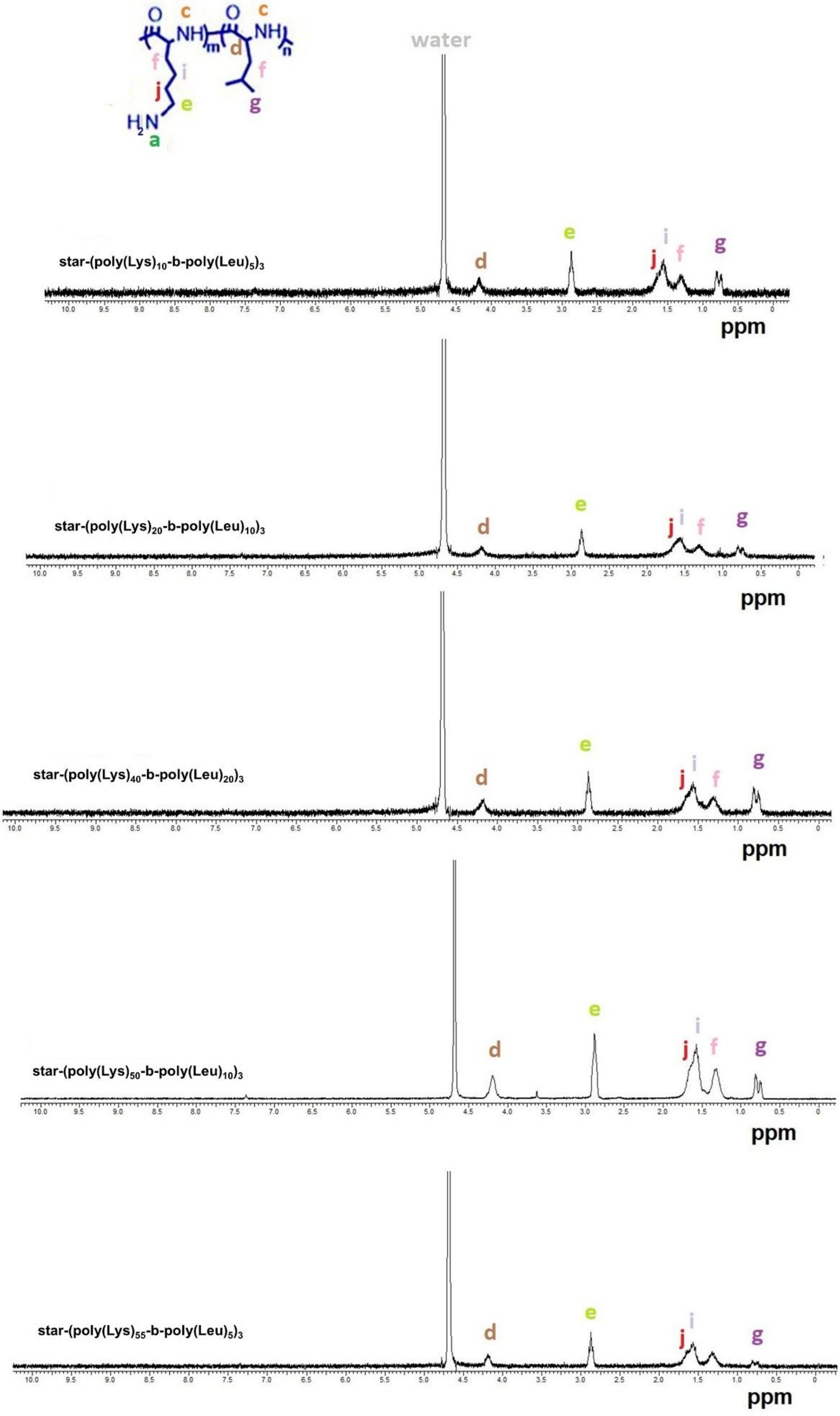

Figure S3. ^{1}H-NMR spectra of the star-shaped deprotected polypeptides in D_2O.

2.3. MALDI-ToF-ToF

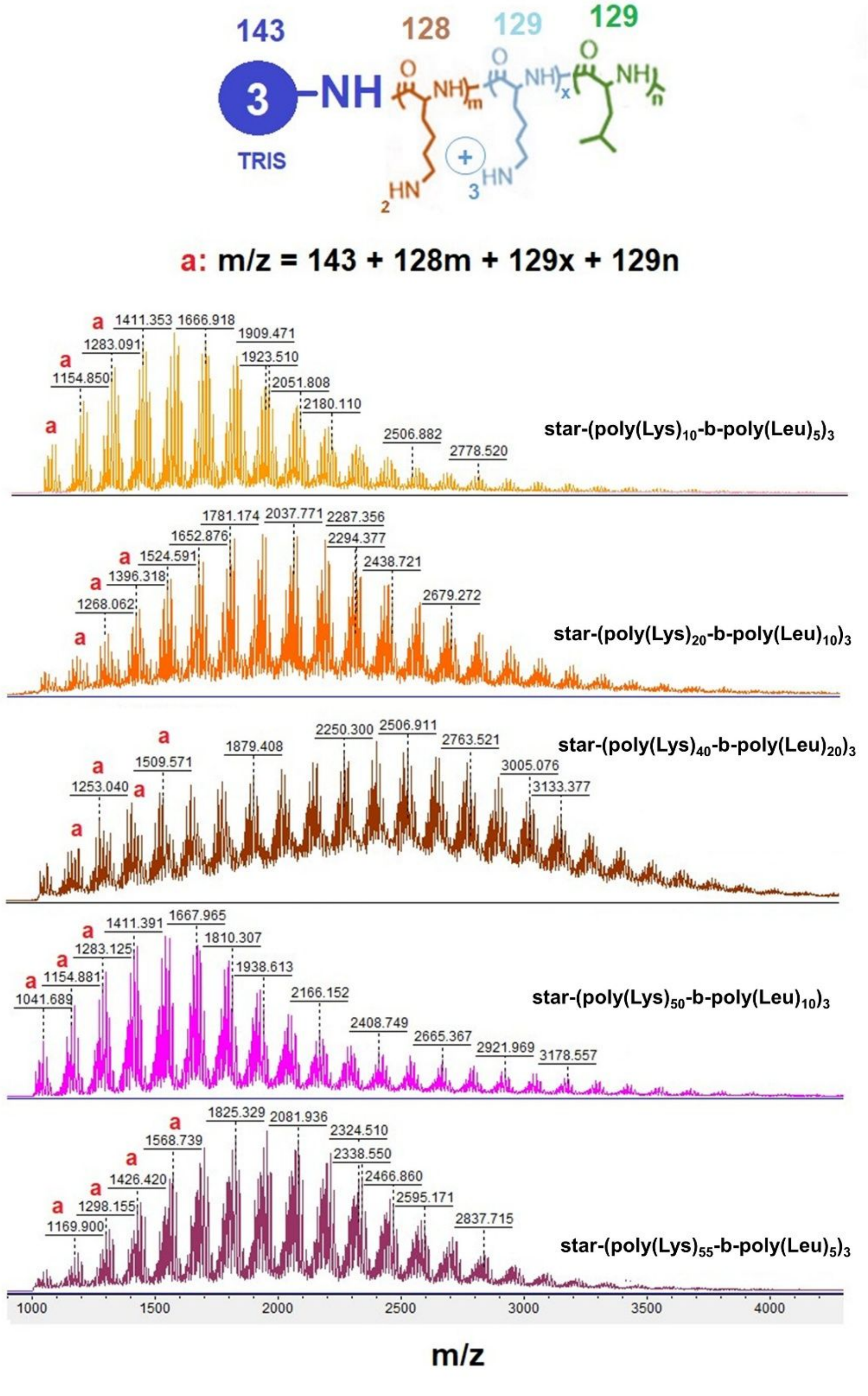

Figure S4. MALDI-ToF-ToF-MS spectra of the star-shaped diblock polypeptides of L-lysine with L-leucine.

2.4. XPS

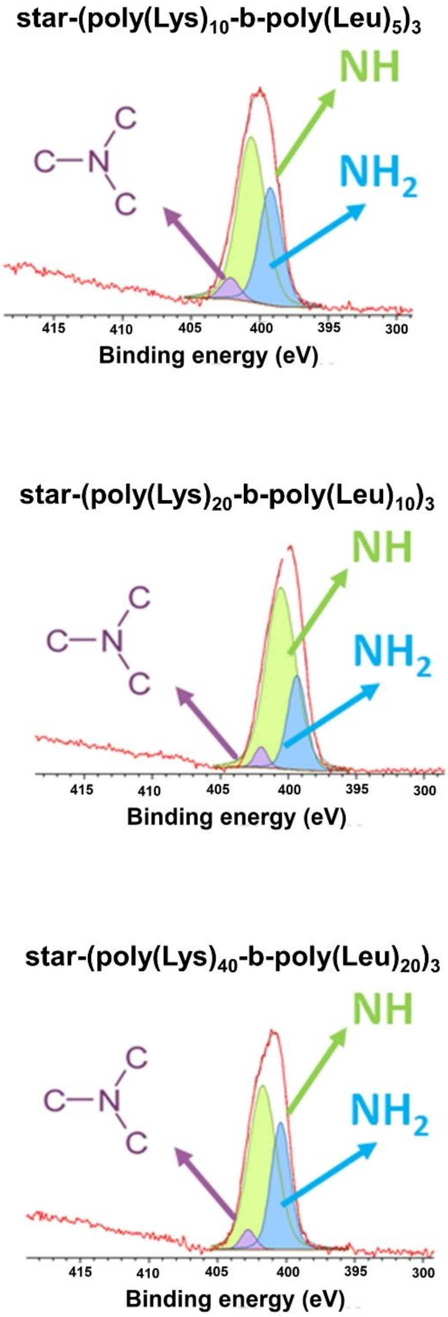

Figure S5. High-resolution XPS (N_{1s} region) for the deprotected star-shaped polypeptides.

2.5. *Thermal analyses*

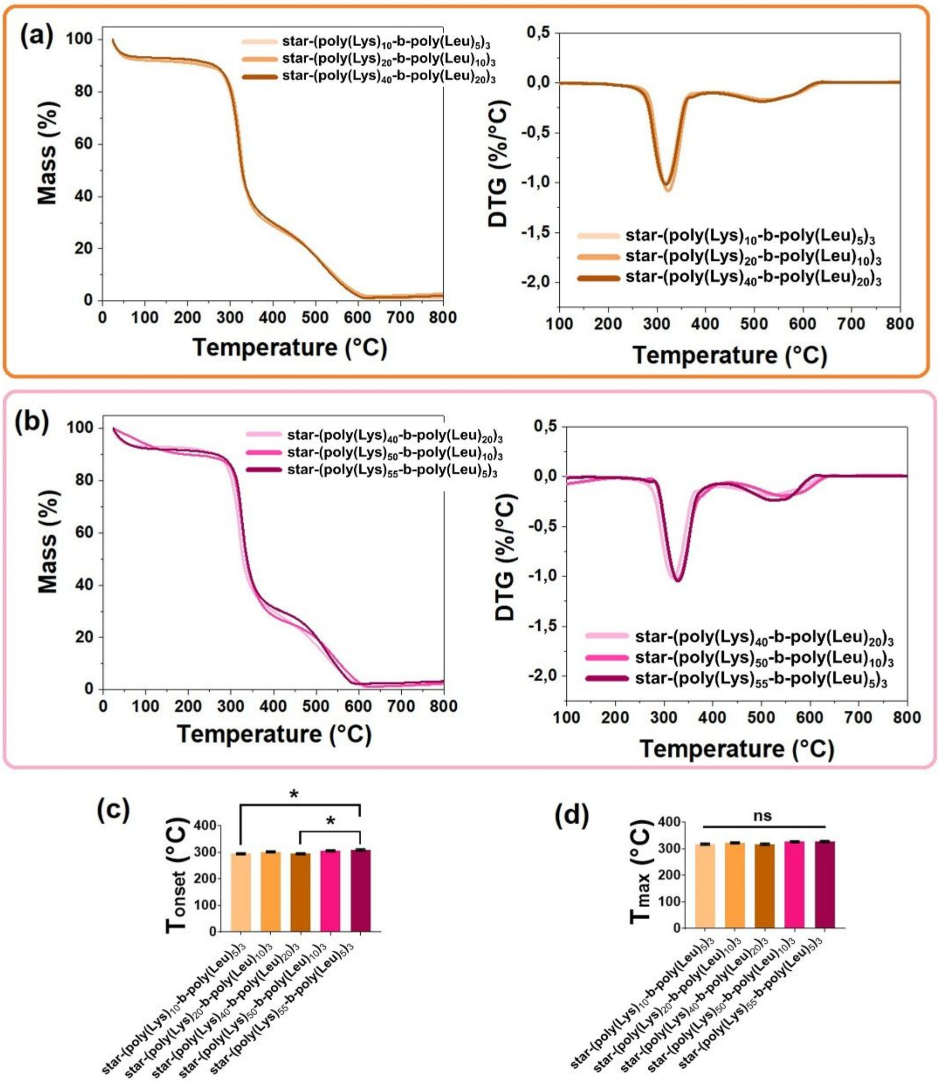

Figure S6. Thermogravimetric curves and thermogravimetric curves of the 1st derivative (DTG) for: (a) star-shaped polypeptides with different amounts of amino acids on their arms and (b) star-shaped polypeptides with different amounts of L-leucine in the hydrophobic blocks. (c) Temperatures of initial thermal decomposition (T_{onset}) and (d) temperatures of maximum thermal decomposition rate (T_{max}) of polypeptides from TGA data. Values are presented as mean ± standard deviation. ANOVA of significant differences between means was determined by Tukey's test and 95% confidence level (*p < 0.05 = significant difference) (ns = non-significant difference, p ≥ 0.05).

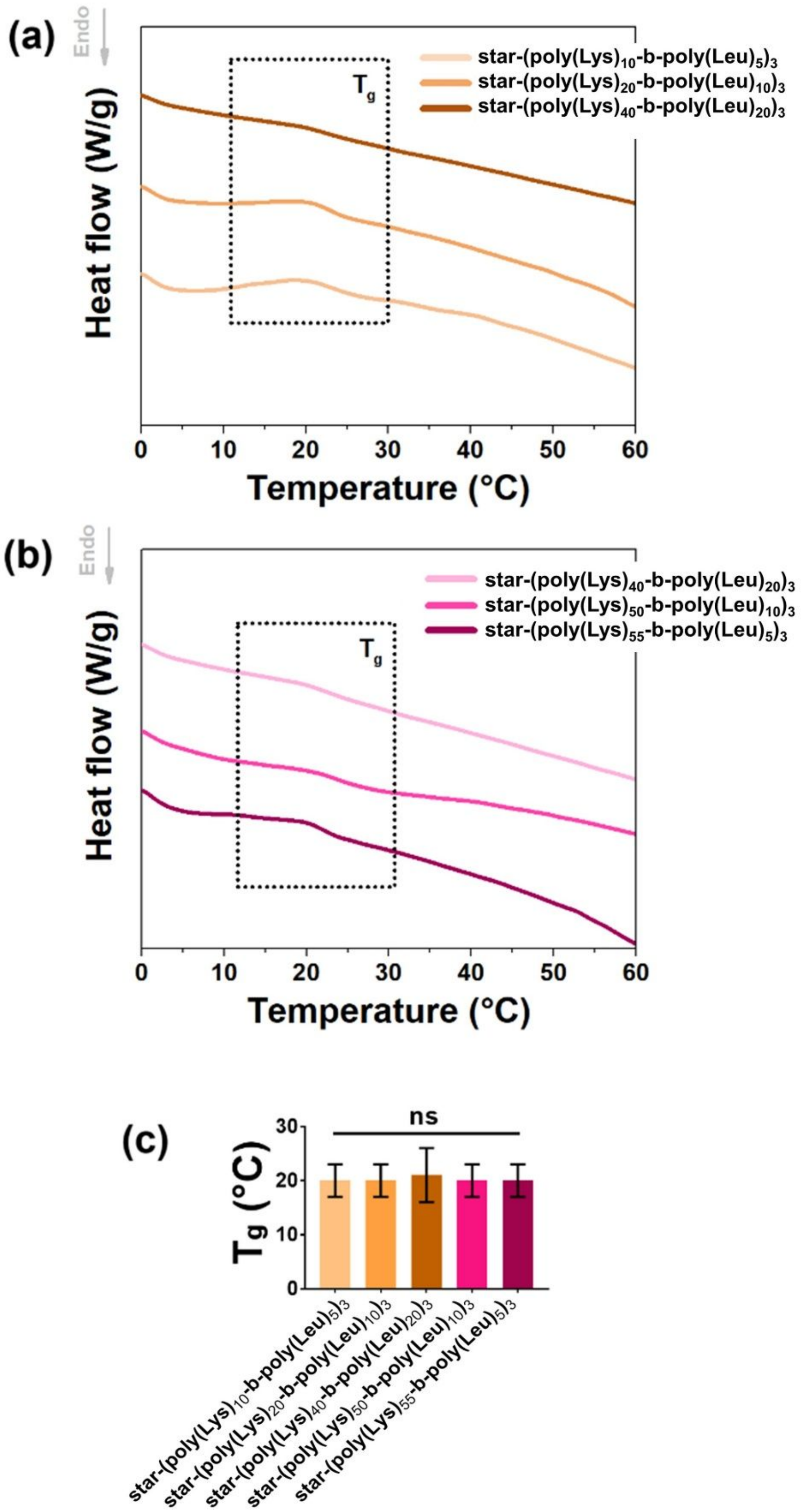

Figure S7. DSC curves for (a) star-shaped polypeptides with different amounts of L-leucine on their arms and (b) star-shaped polypeptides with different amounts of L-lysine on the hydrophobic blocks. (c) Glass transition temperature (T_g) from the DSC data of the polypeptides. Values are presented as mean ± standard deviation. ANOVA of significant differences between means was determined by Tukey's test and 95% confidence level (ns = non-significant difference, $p \geq 0.05$).

2.6. Rheometry

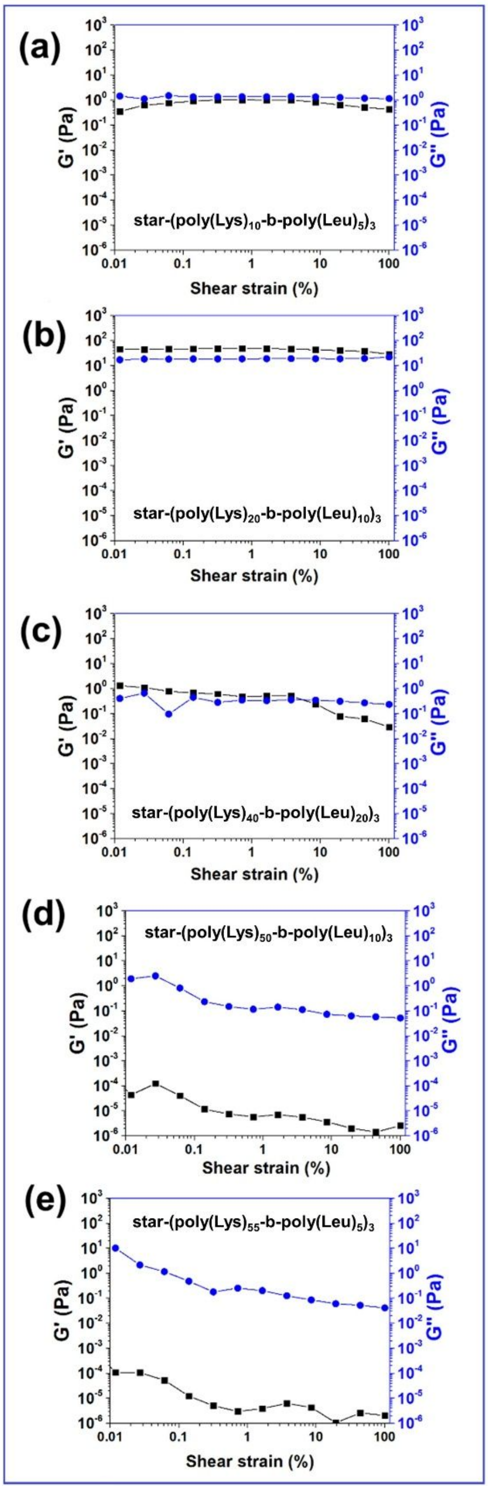

Figure S8. Storage (G') and loss (G'') modulus curves from the polypeptides in water (pH = 7 and temperature = 25 °C) obtained by the oscillatory amplitude sweep test: (a) poly(Lys)$_{10}$(Leu)$_5$, (b) poly(Lys)$_{20}$(Leu)$_{10}$, (c) poly(Lys)$_{40}$(Leu)$_{20}$, (d) poly(Lis)$_{50}$(Leu)$_{10}$, (e) poly(Lis)$_{55}$(Leu)$_5$.

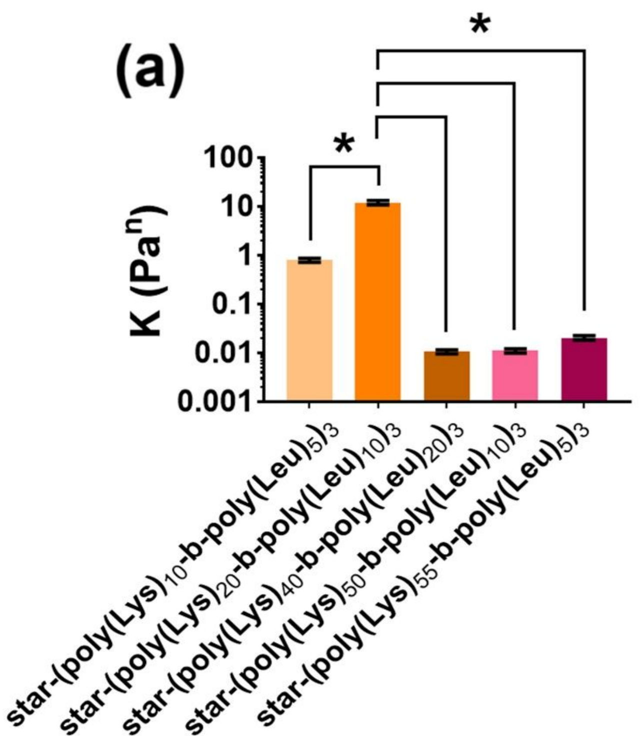

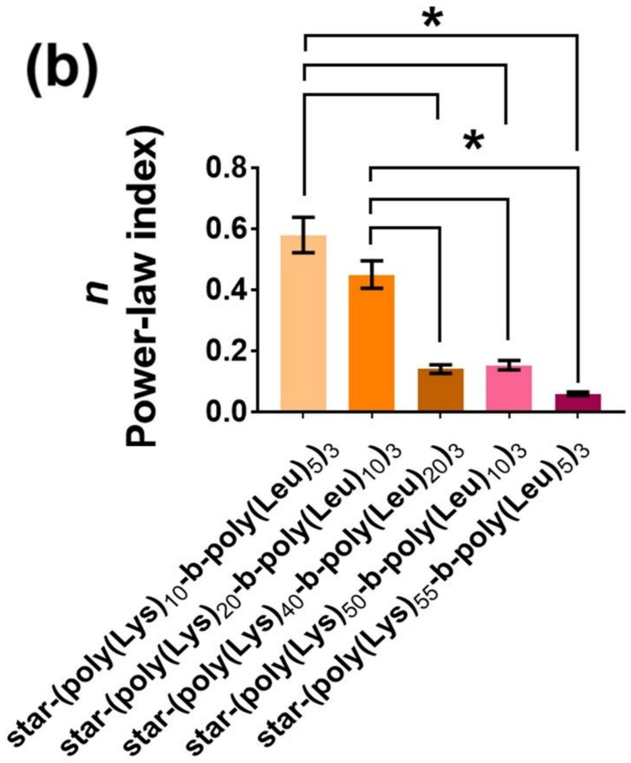

Figure S9. (a) Consistency index (*K*) and (b) power-law index (*n*) from the star-shaped diblock polypeptide solutions (10 wt%) based on L-lysine and L-leucine. Values are presented as mean ± standard deviation. ANOVA of significant differences between means was determined by Tukey's test and 95% confidence level (*$p < 0.05$ = significant difference).

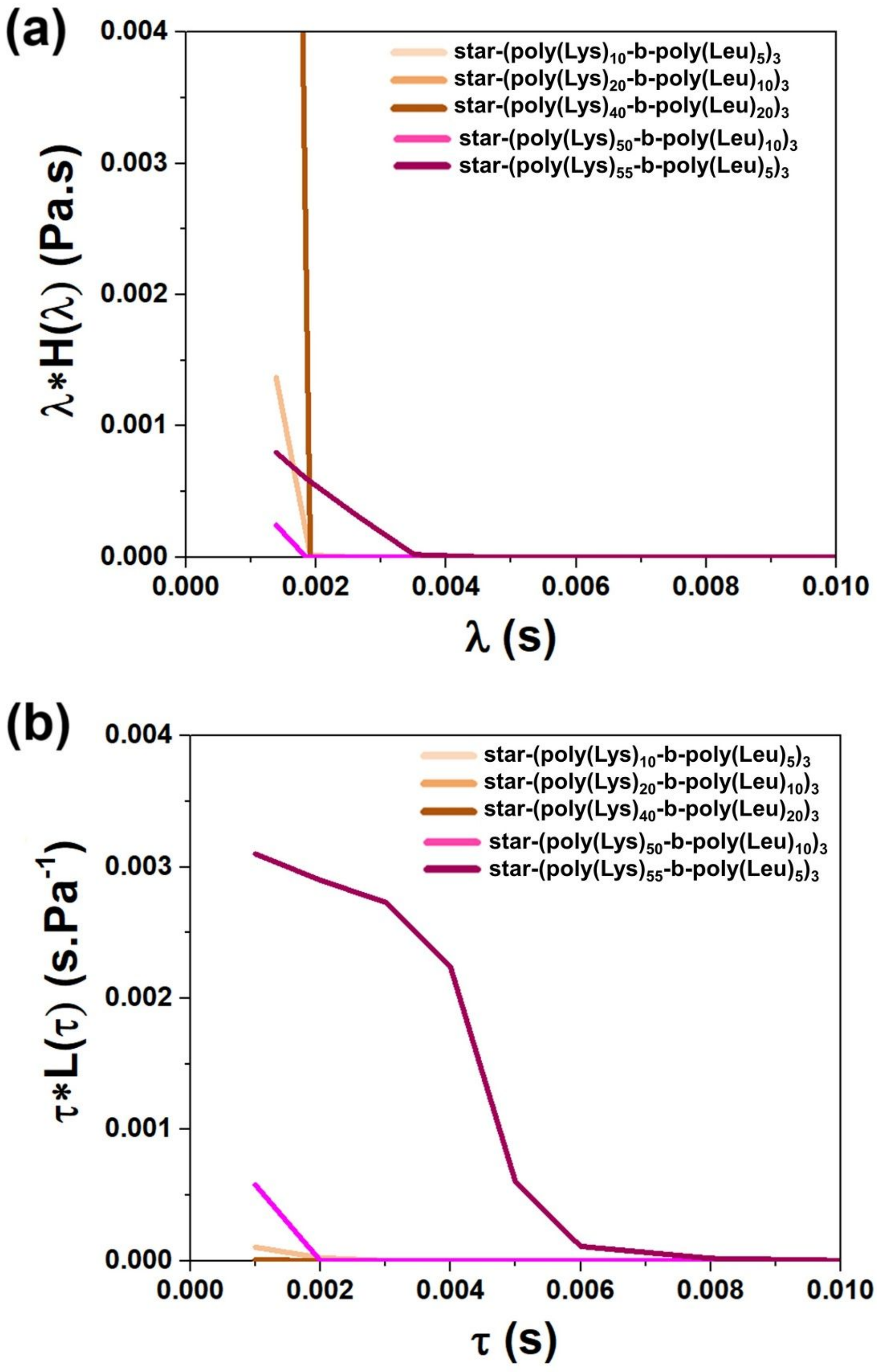

Figure S10. (a) Weighted relaxation spectra and (b) weighted retardation spectra from the star-shaped diblock polypeptide solutions (10 wt%) based on L-lysine and L-leucine.
